# Editorial: An interdisciplinary approach towards a greater understanding of the quality of life in chronic mental illness

**DOI:** 10.3389/fpsyt.2024.1464452

**Published:** 2024-07-26

**Authors:** Fabrizio Stasolla, Laurent Boyer, Bach Tran

**Affiliations:** ^1^ University Giustino Fortunato of Benevento, Benevento, Italy; ^2^ Health Services Research and Quality of Life Center (CEReSS), Aix-Marseille University, Aix-Marseille, France; ^3^ Hanoi Medical University, Hanoi, Vietnam; ^4^ Johns Hopkins University, Baltimore, MD, United States; ^5^ Korea University, Seoul, Republic of Korea

**Keywords:** chronic mental illness, quality of life, interdisciplinary approach, assessment, rehabilitation, interventions

Chronic mental illness entails clinical conditions which may significantly hamper cognitive and emotional functioning, leading to negative outcomes on individuals’ quality of life (QoL). Individuals affected by mental health disorders (e.g., psychiatric symptoms) may encounter daily difficulties to achieve work activities, academic performance, and maintaining interpersonal and social relationships. Because these persons require specific care, families’ and caregivers’ burden is substantially increased. Assessment, preventive, and rehabilitative goals should be considered as a priority accordingly (1–4). Thus, an accurate evaluation is critical for developing a highly customized and tailored intervention to profitably tackle this issue (5, 6). Self-determination, independence, emotional regulation, and constructive engagement should be positively pursued through person-centered programs (7, 8). An interdisciplinary approach is recommended (9, 10). Beside pharmacological treatments, one may envisage cognitive-behavioral therapy, psychosocial strategies, technology-based interventions, and complementary or palliative solutions (11, 12). Eight high-quality papers were included in this Research Topic, concisely detailed below.

Abdulrahman Mahmoud examined the prevalence of depression among science students during Covid-19 pandemic (13). A survey was carried out recruiting 521 participants in Arabia. Results showed that 78% of the sample experienced sadness and depressive symptoms. High scores recorded in the Beck’s Depressive Inventory (BDI) were positively correlated with gender, education, and field of specialty. The BDI scores were additionally associated with academic and demographic variables. Significant symptoms of depression were found among students during the Covid-19 pandemic, warranting psychological counseling for preventive purposes.

Badran et al. explored the QoL for people with psychiatric disorders who were engaged in employment programs (i.e., homogeneous versus heterogeneous) (14). In the homogeneous programs Arabic participants only were involved. In the heterogeneous program both Arabic and Jews. A quantitative study with 104 adults affected by psychiatric disorders was conducted. Participants completed demographic, gender, marital status, religion, and employment questionnaires, next to the Personal Wellbeing Index questionnaire. Two-sample T-Test, exploratory analysis, and multiple linear regressions were performed. Results demonstrated significant differences between programs. Thus, satisfaction of living and health satisfaction were higher in the heterogeneous program. Physical health and gender were the most important variables for explaining QoL in both programs. The family played a crucial role in individual’s employment.

Boumans et al. assessed the self-perceived relationship between creativity experience and mental illness (15). An explorative and interpretative study was designed. Twenty-four professional and semi-professional artists with self-reported experience with mental illness were recruited. Unstructured in-depth interviews were conducted. Transcripts underwent to interpretative analysis guided by a hermeneutic phenomenological frame. Results showed that participants experienced a range of interactions between artistic creativity and mental illness. Three constitutive patterns described that such interactions looked like “flow as a powerful force”, “ambiguous self-manifestation”, and “narrating experience of suffering”. Creativity, mental illness, and their relationship were layered and complex phenomena with different meanings in the individuals’ lives were detailed. Understanding the experience of artists with mental illness could support mental health care.

Ma et al. evaluated the effectiveness of electro-acupuncture in treating post-stroke depression (PSD) by modulating the inflammatory response (16). One hundred and fifty participants with PSD were randomly divided into 75 cases each in the electro-acupuncture group (EA group) and escitalopram group (ESC group). In the EA group underwent 30 sessions were performed. The ESC group received oral escitalopram oxalate tablets for 40 days, plus 30 sessions of sham electro-acupuncture. No statistical differences emerged between groups. Both programs were considered valid for the treatment of mild to moderate PSD.

Lancioni et al. planned a technology-aided intervention (i.e., microswitches linked to a smartphone) to help people with multiple disabilities to control environmental stimulation independently (17, 18). Ten participants were involved. A reversal experimental design was implemented for each participant with baseline and intervention phases. The study assessed whether the participants (a) increased their adaptive responding, and (b) showed signs of satisfaction/happiness. Results evidenced a significant improvement in the adaptive responding for all the participants during intervention phases compared to the baselines. All the participants additionally increased their satisfaction. Such program represents a potentially useful approach to support individuals with multiple disabilities.

Pandiyan et al. investigated the impact of the Covid-19 pandemic on the lifestyle behaviors which included physical activity, sedentariness, healthy eating habits, sleep habits, and tobacco use in persons with and without disabilities in Qatar (19). A cross-sectional study was planned. A structured online questionnaire was used. Data collection included demographic mental health, physical health, eating habits, body weight, sleep and nicotine intake. No differences emerged between people with and without disabilities in the pre-pandemic period. However, during Covid-19 pandemic different perceived changes in mental and physical health, as well as eating habits between individuals with and without disabilities were recorded. A positive relationship was additionally demonstrated between the severity of the disability and the impact of Covid-19 pandemic on the dependent variables. The study confirmed a close relationship between lifestyle and mental health in individuals with disabilities, consistent with previous studies in other populations (20).

Ramesh et al. evaluated the feasibility of the Recovery Assessment Scale – Domains and Stages (RAS-DS) from the perspective of mental health workers (Ref). Participants who had previously sought permission to use the RAS-DS (N=58) completed an online survey aimed at exploring the practicality, acceptability, and applicability of the Scale. The highest-rated feasibility items related to applicability or usefulness in practice, with over the 90% of the sample reporting that the RAS-DS helped “promote discussion” and covered areas that were “meaningful to consumers”. Acceptability items suggested that while the purpose of the RAS-DS was clear, its length was identified as an issue needing resolution. Regarding practicality, the RAS-DS was considered easy to access, but training was deemed necessary to ensure its optimal use.

Saccaro et al. conducted a systematic review and meta-analysis on Moyamoya disease and psychiatric manifestations which included 41 studies. High heterogeneity among the reviewed studies was recorded. Psychosis and neurological symptoms evidenced the highest occurrence. Diagnostic, therapeutic, and prognostic implications of the included studies were critically discussed. The review detailed useful guidelines for pharmacological and psychotherapeutic interventions.

A comprehensive overview of the interdisciplinary approach for improving the QoL in individuals with chronic mental illness was provided, hoping that caregivers, families, clinicians, and researchers may find useful insights and helpful tips for everyday life research and clinical practice. These findings highlight several important factors often neglected such as depression, physical health, lifestyle behaviors and technology-aided interventions.

## Author contributions

FS: Writing – review & editing, Writing – original draft, Conceptualization. LB: Writing – review & editing. BT: Writing – review & editing.
